# Snapshot Serengeti, high-frequency annotated camera trap images of 40 mammalian species in an African savanna

**DOI:** 10.1038/sdata.2015.26

**Published:** 2015-06-09

**Authors:** Alexandra Swanson, Margaret Kosmala, Chris Lintott, Robert Simpson, Arfon Smith, Craig Packer

**Affiliations:** 1 Department of Ecology, Evolution and Behavior, University of Minnesota, Saint Paul, MN 55108, USA; 2 Department of Physics, University of Oxford, Denys Wilkinson Building, Oxford, OX1 3RH, UK; 3 Adler Planetarium, Department of Citizen Science, Chicago, IL 60605, USA

**Keywords:** Community ecology, Population dynamics, Biodiversity

## Abstract

Camera traps can be used to address large-scale questions in community ecology by providing systematic data on an array of wide-ranging species. We deployed 225 camera traps across 1,125 km^2^ in Serengeti National Park, Tanzania, to evaluate spatial and temporal inter-species dynamics. The cameras have operated continuously since 2010 and had accumulated 99,241 camera-trap days and produced 1.2 million sets of pictures by 2013. Members of the general public classified the images via the citizen-science website www.snapshotserengeti.org. Multiple users viewed each image and recorded the species, number of individuals, associated behaviours, and presence of young. Over 28,000 registered users contributed 10.8 million classifications. We applied a simple algorithm to aggregate these individual classifications into a final ‘consensus’ dataset, yielding a final classification for each image and a measure of agreement among individual answers. The consensus classifications and raw imagery provide an unparalleled opportunity to investigate multi-species dynamics in an intact ecosystem and a valuable resource for machine-learning and computer-vision research.

## Background & Summary

Over the last 20 years, camera traps—remote, automatic cameras—have revolutionized wildlife ecology and conservation and are now emerging as a key tool in the broader disciplines of behavioural, population, and community ecology^1^. Historically, cameras have been used to document the presence of rare species in understudied protected areas^2,3^ or estimate densities of individually identifiable animals^4^. But advances in digital technology have increased capacity while lowering prices, resulting in a dramatic increase in the number and diversity of camera trap studies^5^. While traditional analytical approaches for camera trapping data require individually identifiable animals^1,4,6^, recent developments have allowed the expansion of camera trap inference to multiple ‘unmarked’ species^1,7–9^. Although camera-trap surveys are increasing in popularity and scope, they can produce overwhelming amounts of data^10–12^, highlighting the need for efficient image processing techniques. Here we describe the datasets generated by *Snapshot Serengeti*, a large-scale survey that (1) deployed 225 camera traps across a 1,125 km^2^ area in the Serengeti National Park, Tanzania from 2010–2013, (2) used a citizen science website (www.snapshotserengeti.org) to process millions of images, and (3) used a simple algorithm to ensure high reliability of the resultant species classifications.

**Figure 1:**
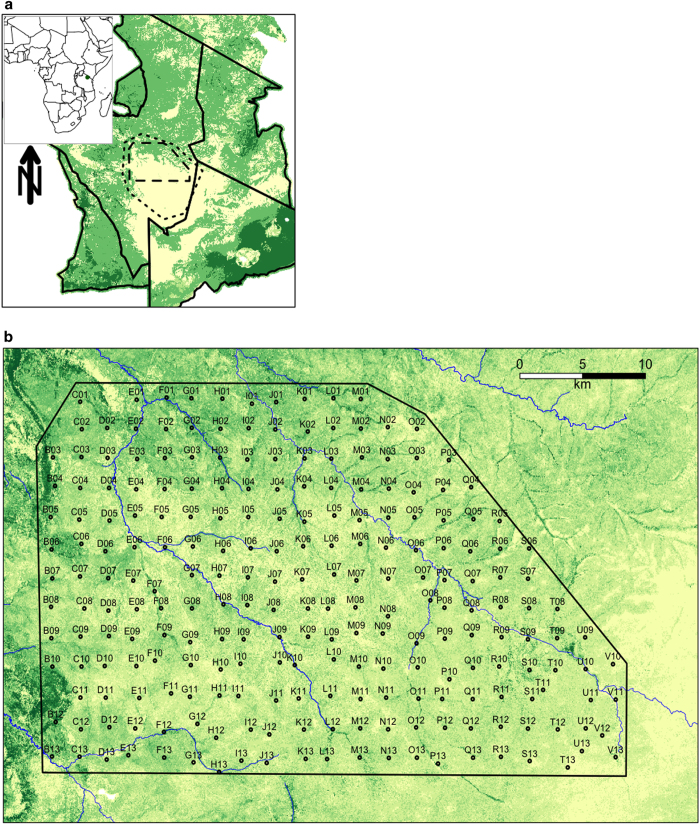
Snapshot Serengeti study area. (**a**) Serengeti National Park. Long-term lion project study area is indicated by dotted line; camera-trap study area is indicated by dashed line. (**b**) Camera trap layout within the long-term Lion Project Study Area. Camera locations are plotted over tree cover (extracted from Landsat imagery), with darker green indicating increased tree cover per 30 m^2^-grid cell.

**Figure 2:**
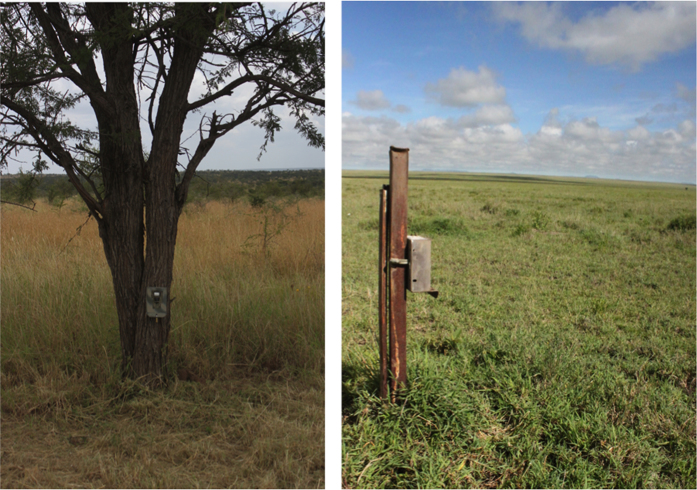
Camera trap placement. Camera traps in steel cases were placed on trees when available (left, *n*=195) and steel poles when no trees were within 1,000 m of the grid-cell centre (right, *n*=35).

We established the *Snapshot Serengeti* camera survey to evaluate spatial and temporal dynamics of large predators and their prey. Serengeti National Park (SNP) is the core of a 25,000 km^2^ savannah ecosystem that straddles the Kenya-Tanzania border in East Africa. The Serengeti is dominated by the annual migration of the combined 1.6 million wildebeest and zebra that follow the seasonal rainfall onto the nutrient rich plains^13^. Since the 1960s, the Serengeti Lion Project has monitored lion population numbers and ranging patterns^14^, and the Tanzania Wildlife Research Institute (TAWIRI) has surveyed major herbivore numbers via flight counts and aerial photography^15–19^. Our camera survey expands upon historical monitoring by providing the first continuous systematic data on all of the larger predator and prey species, day and night, across several years. We set out 225 cameras within a 1,125 km^2^ grid inside the long-term lion study area that covers the intersection of open plains and savannah woodlands (Fig. 1 and Fig. 2), and spans a 1.67-fold rainfall gradient and 1.44-fold productivity gradient (T.M. Anderson, unpublished data.). The camera-trap grid offers systematic coverage of the entire study area (as per O’Brien *et al.*^20^) and ensures at least two cameras per home range for each medium to large mammalian species.

**Figure 3:**
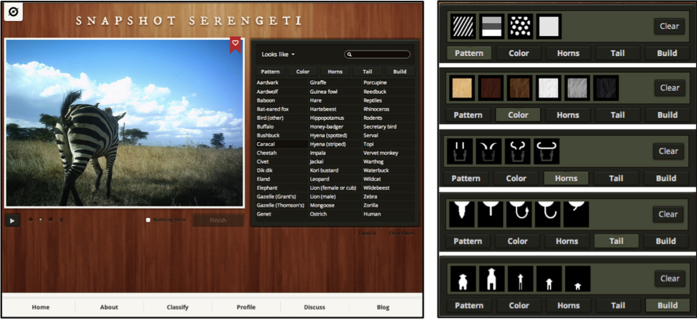
Online interface for www.snapshotserengeti.org. The *Snapshot Serengeti* website interface. The primary interface with all available species options (left) and filters that help narrow users’ choices when classifying species (right).

Between June 2010 and May 2013, the survey operated for a total of 99,241 camera-trap days and produced 1.2 million image sets (each image set contains 1–3 photographs taken in a single burst of ~1 s). In collaboration with The Zooniverse (www.zooniverse.org), the world’s most popular citizen science platform, we developed the website www.snapshotserengeti.org that allowed members of the general public to view and classify each image, identifying species, counting the number of individuals, and characterizing behaviours (Fig. 3).

Every image set was circulated to multiple users to improve data accuracy. More than 28,000 registered users and ~40,000 unregistered users contributed 10.8 million classifications for the 1.2 million image sets. We applied a simple plurality algorithm to produce a ‘consensus dataset’ of final classifications for each image set. The consensus classifications were validated against 4,149 ‘gold-standard’ image-sets that had been classified by experts, revealing 96.6% accuracy for species identifications and 90% accuracy for species counts..

Of the 1.2 million image sets, *Snapshot Serengeti* volunteers indicated that 322,653 contained animals; the remainder were misfires that had been triggered by heat or vegetation. The volunteers identified 48 different species and species groups, including rare and elusive animals such as aardwolf and zorilla (see Table 1).

In this report we describe the field methods, citizen science interface, and consensus algorithm used to produce the following datasets:

(1) Images: Full-resolution images produced by the survey.

(2) Raw classification data: All individual classifications made by all users on all image sets.

(3) Consensus data: Single classification per image set produced by applying the consensus algorithm to raw classifications, along with image metadata (date, time, location).

(4) Operation Dates: Metadata of when each camera was operational

(5) Gold-standard data: Expert classifications for a subset of 4,149 image sets.

We anticipate broad interdisciplinary re-use of these datasets with applications that span basic and applied ecology, citizen-science research, machine learning, and computer vision. The consensus dataset provides species-specific capture histories that can be analysed in a number of ways to evaluate population and community dynamics, either within Serengeti or as part of a larger cross-reserve analysis (see *Usage Notes* for details). The applications for this dataset extend beyond ecological research. For example, increasing the quality of citizen-science data is an area of active research^21–24^. Computer science and informatics researchers can use the raw (un-aggregated) citizen-science answers to develop more complex aggregation algorithms and to test their performance against the gold-standard dataset^25^. Additionally, computer-vision researchers need large human-annotated sets of imagery as training sets in machine-learning algorithms^26^. Our collaborators are currently using this dataset to automate species detection, classification and similar-species differentiation, as well as to develop combined human-machine learning systems and imaging systems for searchable colour. Subsets of the consensus dataset have also been used in classrooms to engage students in authentic research that spans ecology, animal behaviour, and computer science (see *Usage Notes* for examples).

## Methods

### Field methods

We set up an initial camera survey at 200 sites within the long-term Serengeti Lion Project study area from June to November 2010 (Fig. 1a). Cameras were re-installed in February 2011 and have operated continuously thereafter. We expanded the survey from 200 to 225 traps in February 2012 and it is currently on-going. This paper describes data collected until May 2013.

#### Layout

The camera-trap layout placed each camera at the centre of a 5 km^2^ grid cell (Fig. 1b), so as to offer systematic coverage of the entire study area and allow simultaneous monitoring of multiple species^1,20,27–29^. The precise location of each camera was selected as the nearest suitable tree to the pre-determined centre point of each grid cell, which was typically within 250 m of the centre. We selected sites to minimize camera misfires by prioritizing trees that offered shade and by avoiding trees surrounded by tall grass. Where no trees were available within 1 km of the grid cell centre point, we placed cameras on metal poles (Fig. 2).

#### Sites

We set cameras ~50 cm above ground level to capture medium to large vertebrates, housed in steel cases that were attached to trees with 10 cm hardened-steel lag bolts. We trimmed tall grass to <30 cm, and removed low-hanging branches to minimize risk of camera misfires and improve the unobstructed view from the camera. Cameras were pointed to minimise obstructions or risk of misfires rather than with respect to compass direction.

#### Cameras

We primarily used Scoutguard (SG565) incandescent cameras. We initiated the survey using DLC Covert II cameras with an infrared flash, but poor night-image quality prompted the transition to incandescent cameras. The cameras deployed in 2011 included a mixture of DLC Covert Reveal and SG565. Since 2012, all cameras and replacements have been SG565. Animals and weather damaged approximately 15% of cameras annually, requiring repeated replacement.

All survey cameras used passive infrared sensors that were triggered by a combination of heat and motion. Although standard camera-trapping protocols recommend setting sensitivity to ‘high’ for warm climates, this produced unacceptable levels of misfires by the movements of tall vegetation or shadows, thus we set sensor sensitivity to ‘low’ to minimize misfires. The detection radius and field of view were approximately 14 m and 45° for all cameras.

We set all cameras to take 3 photos per trigger in the daytime. At night, infrared-flash cameras took 3 photos per trigger, but incandescent-flash cameras could only take 1 image per trigger due to flash limitations (and occasional camera malfunction created a small number of image sets with varying numbers of images). We refer to each trigger as a ‘capture event’ and the resulting 1–3 images as an ‘image set’; capture events are the units of analysis for ecological studies and comprise the results presented here. We set cameras to ensure at least 1-minute delay between capture events to prevent the memory card being filled to capacity by a single individual or herd.

#### Maintenance

We checked each camera every 6–8 weeks. Except in cases of camera malfunction or damage, this schedule was sufficient to replace batteries and SD cards and ensure continuous operation. We labelled SD cards with the Site ID and the date retrieved and reviewed images in the field to ensure that the camera had functioned properly. We then installed new SD cards and triggered cameras to photograph placards that indicated Site ID, date, and time.

### Data management

We wrote Python scripts to extract date/time from the image files and season, site, and card information from the directory structure. Common errors that arose from camera malfunction (typically due to animal or weather damage) included: the recording of videos instead of still images, incorrect time-stamps for a portion of images, and only 1–2 photos per capture event instead of three. We wrote code in Python, MySQL, and R to flag and correct these errors in the metadata.

### Data processing

#### Platform

We partnered with the online citizen science platform The Zooniverse (www.zooniverse.org) to develop the *Snapshot Serengeti* website (www.snapshotserengeti.org), an online interface where the general public helps process camera trap data. The *Snapshot Serengeti* website utilizes the Zooniverse’s platform *Ouroboros*, written in Ruby on Rails (https://github.com/zooniverse/serengeti). Volunteer classifiers interact with a custom-built JavaScript front-end to classify image sets and results are saved in a MongoDB datastore. Each classification is recorded alongside the time of classification and the identity of the classifier in the form of either a unique identifier assigned by the Zooniverse (for logged in users) or an IP address (for users who have not logged in). *Ouroboros* also allows for custom rules for image-set retirement, as discussed below, and the system can scale rapidly to cope with the demands of a popular site. The interface and images are hosted on Amazon Web Services via Amazon’s Simple Storage Service (S3).

#### Task flow

On the *Snapshot Serengeti* interface (Fig. 3), volunteers identify species in each image set, count the number of individuals, classify behaviour, and indicate the presence/absence of young. For image sets that contain more than one image, volunteers initially see the second image in the set and can toggle between images or use the ‘play’ feature to animate the images. We designed the task flow to help guide people with no background knowledge through the process of identifying the animal(s) in question from 48 possible species and species groups while still providing a rapid route to classification for more knowledgeable participants. Users filter potential species matches by morphological characteristics such as horn shape, body shape, colour, pattern, and tail shape or jump straight to selecting from a list of all species. A ‘nothing here’ button allows users to classify image sets without any animals present. We do not offer an ‘impossible’ or ‘I don’t know’ option because previous testing on a small-scale prototype indicated that such answers were overused and provided no information on the actual species classification, thus wasting volunteer effort. Image difficulty (and probability of being correct) can instead be assessed by measuring variance across individual volunteer answers (see Technical Validation)

#### Circulation and retirement

**Figure 4:**
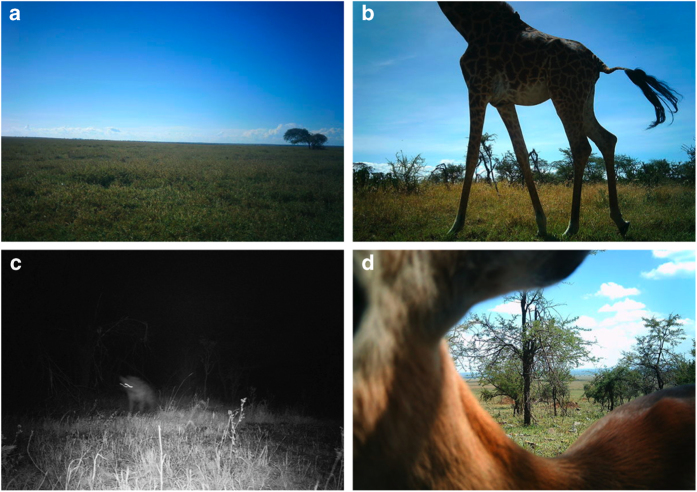
Sample Snapshot Serengeti images. Sample images from image sets retired from Snapshot Serengeti as (**a**) *blank*: receiving five consecutive ‘nothing here’ classifications, (**b**-**c**) *consensus:* receiving 10 matching species classifications, and (**d**) *complete*: receiving 25 classifications regardless of agreement. Note that the plurality algorithm correctly arrived at ‘giraffe,’ ‘spotted hyena,’ and ‘impala’ for images **b**-**d**, respectively (see Tables 2 and 3 for individual classifications).

We circulate each image set to multiple users and retire image sets from circulation when they have met one of the following criteria (see Table 2 and 3 & Fig. 4 for examples):

- *Blank*: the first 5 classifications are ‘nothing here’.
- *Blank_Consensus*: 10 ‘nothing here’ classifications, not necessarily consecutive.
- *Consensus*: 10 matching classifications of species or species combination (e.g., 10 identifications of ‘lion’ or 10 identifications of ‘lion-zebra’); these classifications do not have to be consecutive.
- *Complete*: 25 total non-‘nothing here’ classifications (does not require consensus for any single species).

Note that volunteers classified Snapshot Serengeti data faster than images were produced, and images were re-circulated for classroom use and testing the value of additional classifications. As a result, the number of classifications (11–57 for images containing animals) generally exceeded the number needed for retirement under the above rules.

### Data aggregation

We implemented a simple plurality algorithm to transform the volunteer classifications for each image set into a single aggregated species identification. First, we calculated the number of different species present in an image set as the median number of different species identified across all users for that image set. For all image sets, we assigned the one (or more) species with the most ‘votes’ as the aggregated answer.

We calculated the number of individuals present for each identified species as the median number reported for that image set for that species by all volunteers. We also calculated the proportion of users who chose each behavioural activity or presence of young.

To assess the accuracy of aggregated classifications, we calculated an evenness index, using all non-blank classifications for each image set. When all classifications were in agreement, we assigned the value zero, indicating high accuracy. Otherwise, we used Pielou’s evenness index (Pielou 1966), calculated as $-\left(\Sigma_{i=1}^{S}p_{i}\;\ln\;p_{i}\right)/\ln\;S$, where *S* is the number of different species chosen among all volunteers, and *p*_*i*_ is the proportion of ‘votes’ that species *i* received. The Pielou evenness index ranges from 0 to 1, with 0 indicating low evenness and high accuracy and 1 indicating high evenness and low accuracy. Note that the Pielou evenness index is expected to be high for image sets with multiple species and therefore is not a useful gauge of accuracy in these cases.

### Code availability

#### Classification Interface

The code used to create the Snapshot Serengeti web interface is publicly available at https://github.com/zooniverse/serengeti (current) and archived on figshare^30^.

#### Data processing and consensus calculation

The scripts used to process the data and calculate the consensus classifications are publicly available at https://github.com/mkosmala/SnapshotSerengetiScripts (current) and archived on figshare^31^.

## Data Records

All classification data and metadata are publicly available at Dryad (Data Citation 1).

Images: (all_images.csv; 3,198,737 data rows) URL information for retrieving each image; 1 record per image. All images in this data descriptor can be accessed at https://snapshotserengeti.s3.msi.umn.edu by appending the URL_Info field. For example, appending the value ‘S1/B04/B04_R1/S1_B04_R1_PICT0012.JPG’ yields the full URL: https://snapshotserengeti.s3.msi.umn.edu/S1/B04/B04_R1/S1_B04_R1_PICT0012.JPG. Pasting this value into a browser will display the image in the browser. Note that while we provide all images via the University of Minnesota Supercomputing Institute, this is not a proper archive site. Currently, there are no archiving systems or organizations available for storing the terabytes of images from our study. We hope that image archiving options will become available in the near future.

- *CaptureEventID*: A unique identifier for each capture event and resultant image set.
- *URL_Info*: A URL suffix to be appended to ‘https://snapshotserengeti.s3.msi.umn.edu/’ to yield the full URL of the image.

Raw classification data: (raw_data.csv; 10,530,564 data rows) Raw classification dataset; 1 record per unique user, capture event, and species. Includes images retired as ‘Blank’ and ‘Blank_consensus.’

- *CaptureEventID:* A unique identifier for each capture event and resultant image set.
- *ClassificationID*: A unique identifier for each classification event (one user classifying a single capture event). If a single user identifies multiple species within a capture event, they share the same classification ID.
- *UserID*: Unique user ID for logged-in users; sessionID (unique computer & browser information) for non-logged-in users.
- *Species*: Species selected from a list of 48 options or ‘blank’ for ‘nothing here’
- *Count*: Number of individuals, estimated as 1, 2, 3, 4, 5, 6, 7, 8, 9, 10, 11–50 or 51+.
- *Standing:* Binary indicator of whether any individuals are standing.
- *Resting:* Binary indicator of whether any individuals are resting.
- *Moving:* Binary indicator of whether any individuals are moving.
- *Eating:* Binary indicator of whether any individuals are eating.
- *Interacting:* Binary indicator of whether any individuals are interacting (including both intra- and inter-specific interactions).
- *Babies*: binary indicator of whether young were present.

Consensus classification data and metadata: (consensus_data.csv; 334,671 data rows) Applying the plurality algorithm to the raw classification data yielded a single classification per capture event, accompanied by measures of uncertainty and difficulty. Each species classified in a single capture event receives its own record and species-specific measures of uncertainty. Metadata (data/time & location) are provided to facilitate ecological analyses. This dataset excludes all images retired as ‘Blank’ or ‘Blank_consensus.’

- *CaptureEventID:* A unique identifier for each capture event and resultant image set. Links to CaptureEventID from raw classification data.
- *NumImages:* The number of images in the image set/capture event.
- *DateTime:* The date-time stamp is reported in yyyy-mm-dd hh:mm:ss. Time zone is UTC + 3:00. Note that daylight savings time is not observed in Tanzania.
- *SiteID*: The alpha-numeric site code.
- *LocationX, LocationY:* UTM X and Y coordinates of the site (datum Arc1960, zone 36S)
- *NumSpecies*: The number of species in this capture event.
- *Species*: Species of animal in the capture event (one of 48 possibilities).
- *Count*: Median number of individuals, estimated as 1, 2, 3, 4, 5, 6, 7, 8, 9, 10, 11–50 or 51+.
- *Standing*: The proportion of users who selected this behaviour for this species.
- *Resting:* The proportion of users who selected this behaviour for this species.
- *Moving:* The proportion of users who selected this behaviour for this species.
- *Eating:* The proportion of users who selected this behaviour for this species.
- *Interacting:* The proportion of users who selected this behaviour for this species.
- *Babies*: The proportion of users who selected ‘young present’ for this species.
- *NumClassifications*: The total number of users who classified this capture event.
- *NumVotes*: The total number of users who selected this species for this capture event.
- *NumBlanks:* The number of users who selected ‘nothing here’ for this capture event.
- *Evenness*: The Pielou evenness index of species classifications for the capture event.

Operation dates: (search_effort.csv; 1,128 data rows) The dates that each camera was active and functioning properly, extracted from the image EXIF data as the first and last dates of valid photographs on a given SD card. Valid photographs are defined as those taken while the camera was secured on the tree pointing outwards (as opposed to photos taken after a camera was torn down and facing the ground).

- *Site ID:* The alpha-numeric site code.
- *Start date:* Date of first valid image on a given SD card.
- *End date:* Date of last valid image on the SD card.

Gold standard data: (gold_standard_data.csv; 4,432 data rows) Expert classifications for 4,149 capture events. Note that gold-standard answers are more accurate than answers provided by a single expert because multiple experts reviewed all images for which any single expert expressed uncertainty. In 0.2% of images, the panel of experts agreed that no authoritative species identification could be made; those images are marked as ‘impossible.’

- *CaptureEventID:* Same as in the raw and reduced classification data.
- *NumSpecies:* The number of species in this capture event.
- *Species:* One of the 48 possibilities *or* ‘impossible.’
- *Count*: Number of individuals, estimated as 1, 2, 3, 4, 5, 6, 7, 8, 9, 10, 11–50 or 51+.

## Technical Validation

We asked five researchers with extensive wildlife identification experience to classify 4,149 randomly selected image sets containing animals using the *Snapshot Serengeti* interface; 263 image sets received two expert classifications and 8 image sets received three, for a total of 4,428 classifications. The experts noted whether any image sets were especially difficult or whether they thought the image was identifiable at all. In cases where experts disagreed with the results of the plurality algorithm or had marked an image set as particularly difficult or impossible, AS and CP made the final authoritative identification. Thus, the gold standard dataset included a small number of images that were agreed by multiple experts to be ‘impossible’ to identify. Because the Snapshot Serengeti interface does not allow ‘impossible’ as an option, the consensus answers for these images are incorrect by definition. We compared citizen-science classifications derived from the plurality algorithm with the expert-classified ‘gold standard’ dataset to assess accuracy of species identifications and counts of individuals.

Of the 4,149 image sets viewed by experts, 96.6% of algorithm-derived answers agreed with the expert species classification, though the accuracy rate varied by species (Table 4). Of the 142 image sets in which the algorithm did not agree with the experts, 21% (*n*=30) were marked as ‘impossible’ by experts, 29.5% (*n*=42) reflected cases where the algorithm only identified one of two species identified by experts (for example, only zebra in an image set where both wildebeest and zebra had been present), 3.5% involved cases where the algorithm indicated two species whereas the experts only reported one, and 45.8% (*n*=65) reflected true errors in which the algorithm reported the wrong species. The most common mistakes included misidentification of birds (*n*=11) and incorrectly identifying Grant’s gazelles as Thomson’s gazelles (*n*=11).

Variance in raw classifications strongly predicted whether image sets were classified correctly. Image sets for which the algorithm differed from expert IDs had higher levels of disagreement among raw classifications: the mean evenness score (+/−standard error) was 0.451 (+/−0.004) for correct answers versus 0.725 (+/−0.014) for incorrect answers. Classifications of images that experts identified as ‘impossible’ are considered to be incorrect. We provide guidelines in the *Usage Notes* for using measures of disagreement to measure certainty that a consensus classification is correct and to target image sets for review or exclusion in any given analysis.

For image sets where the plurality algorithm accurately captured all or a subset of species present, we compared the species-specific counts reported by the algorithm to expert classifications (*n*=4,269 species counts). 76.4% of algorithm-derived counts matched expert counts exactly, and 92.98% of algorithm-derived counts were within +/−1 bin of the expert classification (Table 5). Accuracy varied by the number of individuals present: users were > 97% accurate when counting a single individual and least accurate distinguishing between 5–10 individuals.

## Usage Notes

We envision broad applications for these datasets in ecology, informatics, computer vision, and education. Here we provide additional details and guidelines.

### Ecological analyses

The consensus classifications are equivalent to raw data produced by standard camera trapping surveys and include all metadata necessary for applying robust analytical procedures that explicitly consider variations in detection probability. We provide dates of activity for every camera trap, as well as dates, times, and locations for every image. Researchers can thus aggregate camera activity (‘search effort’) and species-specific capture histories into time spans suitable for relative abundance indices^32–34^, single and multi-season occupancy modelling^8,35–38^ across multiple scales^39^, dynamic and multi-species occupancy modelling^1,40–42^, hierarchical binomial or n-mixture models^43,44^. All images are downloadable and identified to species, so capture histories of individually recognized animals can be constructed for species with distinct pelage patterns (e.g., cheetahs, leopards, hyenas, civets, genets, etc.). In such cases, sophisticated mark-recapture analyses can permit spatially-explicit inference^45,46^.

Note that ecological analyses sometimes require higher species-identification accuracy than the 97% returned by the consensus algorithm. Our three ‘certainty’ metrics for consensus answers reflect image difficulty and likelihood of being correct and thus provide guidelines for targeting ‘uncertain’ images for expert review or exclusion from the analysis (see *certainty and difficulty* section below).

### Citizen science and informatics analyses

Crowdsourcing and citizen science are being used increasingly often to produce science datasets^22–24^, but they require robust methods to measure and validate data quality. While our consensus dataset derives from a simple plurality algorithm, more complex algorithms can improve upon these results. For example, Hines *et al.*^25^ weighted raw classifications by individual accuracy, raising overall accuracy to 98%. Our raw classification dataset could be used to develop and test algorithms that employ user-weighting or even apply a Bayesian framework to incorporate information about species likelihood based on previous or subsequent images.

### Computer vision

Object search-and-recognition research requires large data sets of labelled imagery. Reliable data sets of wild animals are rare, due to the enormous task of hand-annotating large numbers of images. By using the raw images together with the consensus dataset, machine-learning algorithms could be developed to automatically detect and identify species, using part of the dataset for training the image-recognition algorithm and the rest for testing the algorithm. Raw images could be used separately, or in conjunction with the consensus data set, to research automatic detection of textures, patterns, and other characteristics of outdoor scenes.

### Education

Higher education teachers can use the consensus dataset and metadata to develop curricula around the scientific method, using charismatic fauna to engage students. As examples, students can ask questions about species abundances and distributions, daily activity patterns and seasonal movements. The dataset can be tailored by the instructor for use by undergraduates for authentic research experiences. (See http://www.cbs.umn.edu/explore/education-outreach/hhmi/projects/nonmajor/serengeti as an example.) This dataset is also suitable for graduate level coursework in ecology, informatics, and computer vision. (See http://rogerioferis.com/VisualRecognitionAndSearch2014/Projects.html as an example.)

#### Dataset and calculation details

The 48 possible ‘species’ options include four ‘group’ categories: *human, bird (other)*, *reptiles*, and *rodents.* ‘Human’ reflects any human activity, including vehicles and hot-air balloons. We made no effort to distinguish among species of hare, jackal, and mongoose. Additionally, lions were split into two categories: *Lion (male)* and *Lion (female & cubs).*

Users typically selected as many behaviours as applicable for a given species in each image but sometimes classified two individuals as displaying two different behaviours by listing the same species twice. For example, an image with one standing zebra and one moving zebra might receive one classification of ‘1 zebra, standing’ and ‘1 zebra, moving’—resulting in multiple classifications of the same species with the same *ClassificationID*. We standardized classifications by combining multiple classifications of the same species within a single *ClassificationID* before applying the consensus algorithm. So ‘1 zebra, standing’ and ‘1 zebra, moving’ were combined to form ‘2 zebras, standing and moving,’ which was the most common classification for such images. Note that the raw classification data set contains separate assessments made by each volunteer and thus does not combine multiple records within *ClassificationID* for any single image.

#### Certainty & difficulty measures

The plurality algorithm produces classifications that are97% correct on average. However, the accuracy varies by species and difficulty and certain analyses may require greater accuracy than obtained from the plurality algorithm. ‘Percent support’ for each species in each image set can be calculated as *NumVotes/NumClassifications* and reflects the proportion of users who identified that species as present in the image set. High values indicate high certainty. High values for *NumBlanks* and *Evenness* (for single species image sets) tend to reflect more difficult image sets for which the consensus vote is more likely to be incorrect. We found that when some users were unsure of their classification, they used the ‘nothing here’ option instead of guessing a species. As a result, difficult images are more likely to have more blank classifications. As described in the Technical Validation, higher *evenness* score reflects lower agreement among classifications and were more likely to be incorrect for single species captures. To increase certainty of datasets for analyses, analyses can be restricted to data that meet any threshold for percent support, *Evenness*, or *NumBlanks*.

## Additional Information

**How to cite this article:** Swanson, A. *et al.* Snapshot Serengeti, high-frequency annotated camera trap images of 40 mammalian species in an African savanna. *Sci. Data* 2:150026 doi: 10.1038/sdata.2015.26 (2015).

## Supplementary Material



## Figures and Tables

**Table 1:** Raw number of capture events for each species as identified by using the plurality algorithm on volunteer classifications.

**Species**	**Total capture events**
aardvark	386
aardwolf	162
baboon	1556
bat eared fox	291
buffalo	13672
bushbuck	252
caracal	79
cheetah	1272
civet	37
dik dik	1483
eland	2689
elephant	10178
genet	27
giraffe	8386
Grant’s gazelle	7723
guinea fowl	7793
hare	398
hartebeest	12431
hippopotamus	2611
honey badger	35
human	9851
impala	8286
jackal	561
kori bustard	688
leopard	228
lion female&cub	3343
lion male	923
mongoose	246
ostrich	673
other bird	5549
porcupine	288
reedbuck	2875
reptiles	131
rhinoceros	30
rodents	48
secretary bird	434
serval	458
spotted hyena	5303
striped hyena	115
Thomson’s gazelle	41420
topi	2299
vervet monkey	314
warthog	7493
waterbuck	353
wildcat	47
wildebeest	100660
zebra	70577
zorilla	17

**Table 2:** Sample classifications for image set retired in Fig. 4 as *consensus* (10 matching classifications)

**ID**	**Species**	**Count**	**Standing**	**Resting**	**Moving**	**Eating**	**Interacting**	**Babies**
*Image set retired as* Consensus—*10 matching species identifications (see Fig. 4b)*								
ASG0010cz5	giraffe	1	N	N	Y	N	N	N
ASG0010cz5	giraffe	1	N	N	Y	N	N	N
ASG0010cz5	giraffe	1	N	N	Y	N	N	N
ASG0010cz5	giraffe	1	N	N	Y	N	N	N
ASG0010cz5	giraffe	1	N	N	Y	N	N	N
ASG0010cz5	giraffe	1	N	N	Y	N	N	N
ASG0010cz5	giraffe	1	N	N	Y	N	N	N
ASG0010cz5	giraffe	1	N	N	Y	N	N	N
ASG0010cz5	giraffe	1	N	N	Y	N	N	N
ASG0010cz5	giraffe	1	Y	N	N	N	N	N
*Image set retired as *Consensus—*10 matching species identifications (see Fig. 4c)*								
ASG0000009	spotted hyena	1	N	N	Y	N	N	N
ASG0000009	jackal	1	N	N	Y	N	N	N
ASG0000009	spotted hyena	1	N	N	Y	N	N	N
ASG0000009	warthog	1	Y	N	N	N	N	N
ASG0000009	spotted hyena	1	Y	N	N	N	N	N
ASG0000009	spotted hyena	1	N	N	Y	N	N	N
ASG0000009	spotted hyena	1	N	N	Y	N	N	N
ASG0000009	spotted hyena	1	N	N	Y	N	N	N
ASG0000009	warthog	1	N	N	Y	N	N	N
ASG0000009	spotted hyena	1	N	N	Y	N	N	N
ASG0000009	wildcat	1	N	N	Y	N	N	N
ASG0000009	spotted hyena	1	N	N	Y	N	N	N
ASG0000009	spotted hyena	1	N	N	Y	N	N	N
ASG0000009	spotted hyena	1	N	N	Y	N	N	N
Each row represents a classification event by a different user.								

**Table 3:** Sample classifications for image set retired in Fig. 4 as *complete* (25 total classifications)

**ID**	**Species**	**Count**	**Standing**	**Resting**	**Moving**	**Eating**	**Interacting**	**Babies**
ASG000xzxd	impala	1	Y	N	N	N	N	N
ASG000xzxd	G. gazelle	1	N	N	Y	N	N	N
ASG000xzxd	reedbuck	1	Y	N	N	N	N	N
ASG000xzxd	reedbuck	1	Y	N	N	N	N	N
ASG000xzxd	dik dik	1	Y	N	Y	N	N	N
ASG000xzxd	*(blank)*							
ASG000xzxd	dik dik	1	Y	N	N	N	N	N
ASG000xzxd	impala	1	Y	N	N	N	N	N
ASG000xzxd	*(blank)*							
ASG000xzxd	reedbuck	1	Y	N	N	N	N	N
ASG000xzxd	*(blank)*							
ASG000xzxd	impala	1	Y	N	N	N	N	N
ASG000xzxd	impala	1	Y	N	N	N	N	N
ASG000xzxd	dik dik	1	Y	N	N	N	N	N
ASG000xzxd	T. gazelle	1	Y	N	N	N	N	N
ASG000xzxd	G. gazelle	1	Y	N	N	N	N	N
ASG000xzxd	G. gazelle	1	Y	N	N	N	N	N
ASG000xzxd	impala	1	Y	N	N	N	N	N
ASG000xzxd	T. gazelle	1	Y	N	N	N	N	N
ASG000xzxd	T. gazelle	1	Y	N	N	N	N	N
ASG000xzxd	dik dik	1	Y	N	N	N	N	N
ASG000xzxd	waterbuck	1	Y	N	N	N	N	N
ASG000xzxd	G. gazelle	1	Y	N	N	N	N	N
ASG000xzxd	G. gazelle	1	Y	N	N	N	N	N
ASG000xzxd	dik dik	1	Y	N	N	N	N	N
Each row represents a classification event by a different user.								
Image set retired as *Complete*—not reaching consensus but having been viewed by 25 people (see Fig. 4d).								

**Table 4:** Accuracy of aggregate classifications of species in 4,149 image sets in comparison with expert classifications

**Species**	* **Correct**	**Total**	**Proportion Correct**
wildebeest	1,727	1,740	0.993
zebra	885	888	0.997
buffalo	244	252	0.968
hartebeest	245	251	0.976
Thomson’s gazelle	197	199	0.990
impala	172	181	0.950
other bird	101	134	0.754
warthog	120	120	1.000
giraffe	92	92	1.000
elephant	84	85	0.988
human	72	75	0.960
Grant’s gazelle	55	67	0.821
guinea fowl	55	56	0.982
spotted hyena	55	55	1.000
hippopotamus	28	28	1.000
eland	23	25	0.920
reedbuck	22	25	0.880
baboon	22	22	1.000
topi	15	20	0.750
lion (female&cubs)	18	18	1.000
dik dik	8	8	1.000
porcupine	8	8	1.000
cheetah	6	6	1.000
mongoose	5	6	0.833
serval	6	6	1.000
aardvark	4	4	1.000
bushbuck	3	4	0.750
kori bustard	4	4	1.000
secretary bird	4	4	1.000
jackal	1	3	0.333
leopard	3	3	1.000
ostrich	3	3	1.000
vervet monkey	3	3	1.000
aardwolf	1	2	0.500
hare	1	1	1.000
lion (male)	1	1	1.000
rhinoceros	1	1	1.000
rodent	0	1	0.000
waterbuck	1	1	1.000
*Total* indicates the number of image sets containing that species, as identified by experts			

* *Correct* indicates the subset of those image sets for which the plurality algorithm arrived at the correct answer. *Proportion Correct* is given by *# Correct / Total.* Note that the 30 image sets marked as ‘impossible’ by experts do not appear in this table. Twenty-nine image sets were marked impossible due to insufficient amount of detail to make a definitive identification; one image set was marked impossible because it contained an animal (*duiker*) not in the list of 48 species available to the volunteers.

**Table 5:** Validation of species counts against expert classifications for image sets with a single species

**Species Counts**	* **Validated**	**Proportion Exactly Correct**	**Proportion within+/−1 bin**
1	1,662	0.975	1.000
2	626	0.827	1.000
3	401	0.721	0.965
4	309	0.621	0.942
5	213	0.507	0.897
6	202	0.386	0.797
7	136	0.397	0.794
8	98	0.286	0.653
9	84	0.298	0.726
10	54	0.185	0.704
11–50	456	0.713	0.776
51+	16	0.438	0.938

* *Validated* is the total number of counts validated by experts. *Proportion Exactly Correct* reflects the proportion of algorithm-derived counts that matched expert classifications exactly. *Proportion within* +/−*1* reflects the proportion of algorithm-derived accounts that fell within 1 count bin above or below the expert classification.
